# Female house sparrows "count on" male genes: experimental evidence for MHC-dependent mate preference in birds

**DOI:** 10.1186/1471-2148-11-44

**Published:** 2011-02-14

**Authors:** Matteo Griggio, Clotilde Biard, Dustin J Penn, Herbert Hoi

**Affiliations:** 1 Konrad Lorenz Institute for Ethology, Austrian Academy of Sciences, Austria; 2 Konrad Lorenz Institute for Ethology, Department of Integrative Biology and Evolution, University of Veterinary Medicine Vienna, Savoyenstrasse 1a, 1160 Vienna, Austria; 3 UMR 7625 Ecologie & Evolution, Université Pierre et Marie Curie UPMC, CNRS ENS AgroParisTech, 7 quai Saint Bernard (case 237), F-75252 Paris Cedex 05, France

## Abstract

**Background:**

Females can potentially assess the quality of potential mates using their secondary sexual traits, and obtain "good genes" that increase offspring fitness. Another potential indirect benefit from mating preferences is genetic compatibility, which does not require extravagant or viability indicator traits. Several studies with mammals and fish indicate that the genes of the major histocompatibility complex (MHC) influence olfactory cues and mating preferences, and such preferences confer genetic benefits to offspring. We investigated whether individual MHC diversity (class I) influences mating preferences in house sparrows (*Passer domesticus*).

**Results:**

Overall, we found no evidence that females preferred males with high individual MHC diversity. Yet, when we considered individual MHC allelic diversity of the females, we found that females with a low number of alleles were most attracted to males carrying a high number of MHC alleles, which might reflect a mating-up preference by allele counting.

**Conclusions:**

This is the first experimental evidence for MHC-dependent mating preferences in an avian species to our knowledge. Our findings raise questions about the underlying mechanisms through which birds discriminate individual MHC diversity among conspecifics, and they suggest a novel mechanism through which mating preferences might promote the evolution of MHC polymorphisms and generate positive selection for duplicated MHC loci.

## Background

Darwin suggested that female choice can help explain the evolution of extravagant secondary sexual characters in males, but he struggled over how to understand why females evolve mating preferences for such males [1]. Jerram Brown decided to "put aside the idea that there is a best male and that he is best for every female," and instead, he argued that females should prefer genetically compatible or heterozygous males to increase offspring heterozygosity or genetic diversity [2] (also see [3-5]). He was inspired by studies on house mice (*Mus musculus*) that found disassortative mating preferences for genes of the major histocompatibility complex (MHC) [6,7]. MHC genes are a multigene family in vertebrates that encode cell-surface glycoproteins (class I and II molecules) that control antigen presentation to T-lymphocytes, and through this mechanism MHC genes play a pivotal role in immune recognition of pathogens and parasites. MHC-disassortative mating preferences may function to increase offspring heterozygosity - MHC or genome-wide - as both can enhance resistance to infectious diseases [8-12]. Furthermore, MHC-disassortative mating preferences can also help to explain the extraordinary polymorphism of MHC genes [13]. More recent studies have found MHC-dependent mating preferences in fish [14-17], reptiles [18], and primates and other mammals [19-21]. However; more studies are needed, especially in birds and other wild, outbred species [22-25]. Our aim was to test whether (and how) MHC genes influence mating preferences in house sparrows (*Passer domesticus*).

Several observational studies suggest that MHC genes play a role in mate choice in birds. First, a study on pheasants (*Phasianus colchicus*) suggests that females prefer males with "superior" disease-resistant MHC-genotypes, as predicted by good genes models of sexual selection [26]. Second, a study in Savannah sparrows (*Passerculus sandwichensis*) found evidence that females avoid males sharing similar MHC alleles [27]. Third, a study on house sparrows found evidence that females avoid mating with males that have low individual MHC diversity and males that are too dissimilar (no common alleles) at MHC (class I) loci [28]. Fourth, a study on Seychelles warblers (*Acrocephalus sechellensis*) found that females were more likely to have extra-pair offspring when their social mate had low MHC diversity, and the MHC diversity of the extra-pair male was higher than that of the cuckolded male [29]. Finally, MHC genes may also play a role in cryptic mate choice, as suggested in studies on fish, birds and mammals [21,30-33]. For example, it has recently been found that in peacocks (*Pavo cristatus*), females lay more eggs when mated with males with high individual MHC diversity [32]. Moreover, in red jungle fowl (*Gallus gallus*), males invest less sperm when copulating with females carrying similar MHC alleles [33]. Taken together, observational studies in the wild and experimental studies on cryptic mate preferences provide intriguing evidence that MHC genes influence mating preferences in birds.

MHC-dependent mating preferences might function to enhance offspring heterozygosity, or produce offspring with intermediate or optimal levels of MHC-heterozygosity [13]. The "optimal heterozygosity" hypothesis follows from models suggesting that expressing more MHC molecules during thymic selection has negative effects on the development of the T cell repertoire [13]. Interestingly, this hypothesis is directly supported by studies on stickleback fish (*Gasterosteus aculeatus*): individuals vary in the number of MHC alleles they carry (due to variation in heterozygosity, number of loci, or both), and females with a low number of MHC alleles prefer males with a high individual diversity, whereas females with high diversity prefer males with low individual diversity ("allele optimization strategy") [15]. Thus, in sticklebacks, females' preferences are based on the *number *rather than the *similarity *of alleles they share with prospective mates [34], and this preference is functional because individuals with an intermediate number of MHC alleles are the most resistant to parasites [35,36]. Unlike disassortative mating, however, it is unclear how such sexual selection for optimizing offspring heterozygosity can explain or contribute to the evolution of MHC polymorphisms [37]. Therefore, it is still unclear whether MHC-dependent mating preferences provide a general explanation for the evolution of MHC polymorphisms, or not.

**Figure 1:**
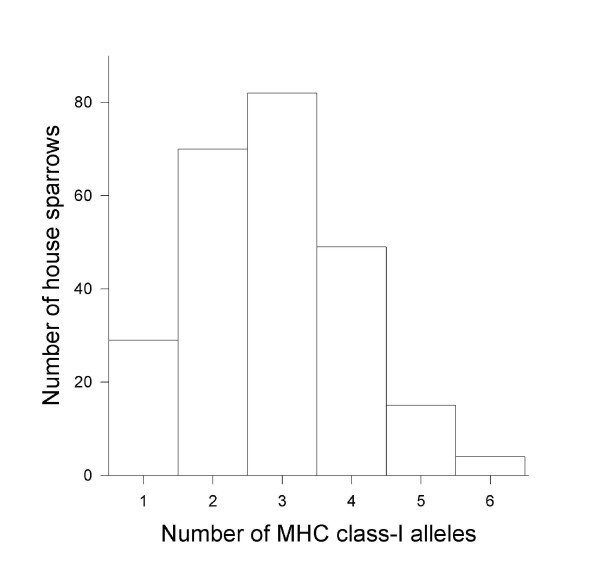
**Frequency distribution of the number of MHC class I alleles in the 249 house sparrows captured from an Austrian population and used in the experiment**.

We specifically tested whether female house sparrows are attracted to males carrying a high allelic diversity at MHC loci (good genes) [29], or whether their preferences maximize or optimize MHC allelic diversity of offspring (genetic compatibility) [16,18]. If females seek the "best" mating partner, one would predict that most females will prefer one or few males, but if they are searching for a genetically compatible partner, females will differ in their preferences of males based on their own MHC diversity. We conducted a female mate preference test using a four-choice apparatus, in which the females had a choice between three males, each having either with low (1-2 alleles), medium (3 alleles) or high (4-6 alleles) number of MHC class I alleles (LM, MM and HM groups respectively), or a female control (CF) in a fourth chamber (for MHC alleles distribution in the population see Figure 1). To estimate proximity preference, we measured the time spent by each female on the part of the perch in front of a male's compartment (choice time). Stimulus individuals were tested to three experimental groups of focal females: females with low (LF), medium (MF) and high (HF) diversity (number) of MHC class I alleles.

## Results

**Figure 2:**
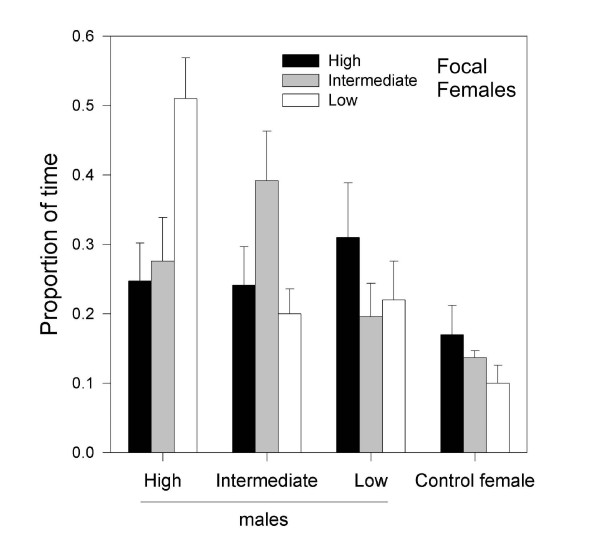
**Percentage of time spent by focal females in the choice area (mean time ± 1 SE) of stimulus individuals (three males and a control female), according to their individual MHC diversity**.

Since focal females spent the lowest proportion of their time in front of the control female chamber, this confirmed that females showed sexual and not merely social preferences (preference for males over stimulus females, ANOVA test: F _1,214 _= 15.15, *P *< 0.001; see Figure 2). We did not detect an overall preference for males with either low, medium or high MHC diversity, but when we considered the individual MHC allelic diversity of the females, we found that low diversity females spent significantly more time in front of the high diversity male (i.e. HM; GLMM analysis for 54 trials: female group: F _2,130 _= 0.45, *P *= 0.64; stimulus group: F _2,130 _= 2.11, *P *= 0.13; female group*stimulus group: F _4,130 _= 3.82, *P *= 0.006; Figure 2, see Table 1 and Table 2). We found no evidence that morphological traits or multi-locus heterozygosity (6 microsatellite markers) had an effect on females' preferences (for more details see Table 1). Lastly, we found no significant correlations between MHC genotypes and variation in morphological traits (wing and tarsus length, body mass and black breast patch size, all F _6,155 _< 1.40 and *P *> 0.1).

**Table 1:** Generalized linear mixed model investigating variation in female mate preferences.

Factors	**d.f**.	*F*	*P*
Female group	2, 130	0.450	0.639
Stimulus group	2, 130	2.108	0.126
Male wing length	1, 130	0.012	0.915
Male tarsus length	1, 130	0.227	0.635
Male body mass	1, 130	0.042	0.838
Male badge size	1, 130	0.274	0.601
Stimulus individual heterozygosity	1, 130	0.040	0.842
Female group × Stimulus group	4, 130	**3.824**	**0.006**
Female group × Stimulus individual heterozygosity	2, 130	0.121	0.886
Female group × Male badge size	2, 130	0.489	0.614

Fixed effects were female group (High, Intermediate and Low number of MHC alleles) and stimulus group (Males with High, Intermediate, Low number of alleles, and the control group). Male wing length, tarsus length, body mass, badge size and heterozygosity were entered into the model as covariates. We fitted the female individual identity as a random factor (F _1,130 _= 0.031, *P *= 0.86) to control for the non-independence of the data. Significant *F *and *P*-values are shown in bold.

**Table 2:** GLM post hoc test (Tukey honestly significant difference test) for the effect of the interaction between female group and stimulus group on female mate preference (see Table 1).

Focal females	Stimulus groups			Difference	*P*
Low	High	vs	Control	**0.452**	**< 0.001**
			Intermediate	**0.316**	**0.003**
			Low	**0.347**	**0.001**
	Intermediate	vs	Control	0.136	0.386
			Low	0.030	0.984
	Low	vs	Control	0.105	0.603

Intermediate	High	vs	Control	0.165	0.484
			Intermediate	0.102	0.812
			Low	-0.139	0.623
	Intermediate	vs	Control	0.063	0.946
			Low	-0.241	0.170
	Low	vs	Control	0.304	0.052

High	High	vs	Control	0.084	0.870
			Intermediate	-0.091	0.842
			Low	0.004	1.000
	Intermediate	vs	Control	0.175	0.392
			Low	0.095	0.824
	Low	vs	Control	0.803	0.885

The difference refers to the mean difference in the arcsin transformed percentage of time females spent close to the stimulus groups. Significant values are shown in bold.

## Discussion

We found no significant evidence that females prefer males carrying a particular number of MHC alleles, as predicted by the good genes hypothesis [26]. However, we found that females discriminate among males carrying different levels of individual MHC allelic diversity, and females' preferences depend upon their own and the individual diversity of potential mates. More specifically, females with a low number of alleles spent significantly more time near males carrying a high number of alleles, which might reflect a "mating up" tactic. Unlike stickleback fish [15], however, females with intermediate or high number of alleles did not show any significant preference based on males' MHC diversity. To our knowledge, our results provide the first experimental evidence in birds that MHC genes play a role in mating preferences. It is unclear whether our findings predict actual mating patterns in the wild, although they are consistent with an observational study on a wild population of house sparrows that found evidence that females avoid mating with males with low individual MHC diversity [28].

There are several reasons to suspect that this *mating-up preference by allele counting *might enhance offspring disease resistance and fitness. First, a previous study on house sparrows found that mating pairs with high individual MHC diversity had offspring with high individual diversity [28], which suggests that females with low diversity can increase individual (and brood) diversity of their offspring by mating up. Second, another study with house sparrows found MHC-dependent immune responses (assayed with phytohemagglutinin and sheep red blood cells) [38], and although the number of individual MHC alleles had no detectable effect, a study on peacocks found greater immune responses to phytohemagglutinin with increased individual MHC diversity [32]. Third, an experimental study with stickleback fish indicates that there is an optimal number of individual MHC alleles for mounting immune defenses against multiple parasites [34], which means that females with low individual MHC diversity should increase offspring disease resistance by mating with males carrying high diversity. Nevertheless, further studies are needed to determine the expression of MHC in sparrows and to understand how MHC allele number affects host immune resistance.

Moreover, our findings are consistent with recent studies indicating that females' quality or condition influences their mating preferences [39-42]. For example, female house mice show odour preferences for outbred over inbred males, though only inbred females show this preference [43]. House sparrows, in fact, provide another example of such condition-dependent preferences [44]. The black throat patch (badge) of the males is an intensively studied plumage trait that appears to be involved in female mate choice, though differences exist among populations. A recent study found that females in poor body condition, unlike those in good condition, preferred males with average-size badges. Taken together, our results here are consistent with the idea that females' mating preferences vary depending upon their own quality.

## Conclusions

After dividing females according to their individual number of MHC alleles, we found that females with a low number of alleles are most attracted to males carrying a high number of MHC alleles, which might reflect a mating-up preference by allele counting. Our findings raise questions about the phenotypic cues sparrows utilize to assess MHC diversity among conspecifics and the evolutionary consequences of these preferences. We found no evidence that individual MHC diversity was associated with any phenotypic trait (body size or size of ornaments) (see also [28]). It has been widely assumed that birds are microsmatic or anosmatic; however, there is increasing behavioral, physiological as well as genetic evidence that their olfactory abilities are better than generally assumed (reviewed in [22,45]), raising the possibility that some birds might utilize olfactory cues to assess potential mates. Indeed, T-maze experiments have found that crested auklets (*Aethia cristatella*) exhibited an attraction to conspecific feather odour and preferentially orientated towards two chemical components of feather scent [46]. Using a similar apparatus, it was demonstrated that blue petrels (*Pachyptila desolata*), could discriminate between their own, their mate's and an unknown conspecific's odour, and were attracted to their mate's odour [47]. Moreover, it was found that the volatile compounds in the preen oil (preen gland secretions) of a songbird, the dark-eyed junco (*Junco hyemalis*), contain reliable information about individual identity, sex and population of origin [48]. Thus, it is plausible that MHC-dependent mate choice in birds is mediated by olfactory mechanisms. It remains to be seen whether mating with males having high individual MHC diversity provides indirect benefits for low diversity females. Similarly, it has been suggested that homozygous females have the most to gain by mating with heterozygous males (for a review see [49]). Finally, our findings suggest that mating preferences can potentially provide a selective factor favoring MHC allelic diversity in populations, duplication of MHC loci and copy number variation. Duplications that increase the number of MHC loci must eventually have negative consequences on individual immunity, but the selective forces shaping the number and diversity of MHC loci within a species may include mating preferences tracking individual immunological optima, which likely varies in time and space.

## Methods

### Subjects and housing

Males and female house sparrow were collected at the Vienna Zoo (47°56'N, 16°45'E), Cobenzl (48°16'N, 16°19'E) and Feuersbruun (48°26'N, 15°47'E) in the winter preceding the experiment. A total of 54 focal females and 156 stimulus males and 39 stimulus females were housed outdoors in seventeen aviaries, in which they were attributed at random (aviary size: 3.5 m × 3.5 m × 3 m; about fifteen individuals per aviary). All birds were over 1 year old. All aviaries were equipped in the same way with vegetation, several perches (about seven per aviary). Commercial food for granivorous passerines and water were provided *ad libitum*. The initiation of breeding immediately after the experiment and several successful breeding attempts suggest that the housing conditions and experiment were appropriate and had no negative effect on the birds' health or condition.

### MHC characterization (DNA isolation, PCR and SSCP)

Genomic DNA was extracted from blood samples with a DNA extraction kit (DNeasy Blood and Tissue Kit, QIAGEN GmbH) according to the manufacturer's protocol. We estimated the overall number of MHC alleles per individual by amplifying exon 3 of a class I locus, which corresponds to the peptide-binding region (PBR) [50-52]. PCR amplifications were performed using a fluorescent (6'-FAM) labelled primer (23 M- GCG CTC CAG CTC CTT CTG CCC ATA) and an unlabeled primer (A21M- GTA CAG CGG CTT GTT GGC TGT GA) [50,51]. The PCR amplification (T1 thermocycler, Biometra) contained a final volume of 25 μL, which included 50 to 100 ng of genomic DNA, 0.6 μM of each primer and 12.5 μL Multiplex PCR Kit (QIAGEN GmbH) (containing hot-start DNA polymerase, PCR buffer and dNTP mix). The PCR program began with 15 min initial heating at 95°C followed by 35 cycles of 30 s denaturation at 94°C, 35 s annealing at 64°C and 90 s extension at 72°C. A final elongation step was run at 72°C for 10 min. To control for PCR artifacts, we used a high-end polymerase and 2 step negative controls (for both the PCR and capillary sequencer).

MHC diversity was screened using capillary electrophoresis single strand conformation polymorphism (CE-SSCP) [50,51,53]. The fluorescent-labelled PCR samples were prepared for electrophoresis by combining 1 μL PCR product with 14 μL loading mix (13.5 μL Hi-DI formamide, 0.5 μL of in-house prepared ROX size standard, [54]). The mixture was heated for 3 min at 95°C to separate the complementary DNA strands, chilled on ice for 4 min and analysed by capillary electrophoresis (ABI PRISM 3130 xl automated DNA Sequencer, Applied Biosystems). The CE-SSCP polymer consisted of 5% GeneScan polymer (Applied Biosystems), 10% glycerol, 1xTBE, and HPLC-water. The running buffer mixture contained 10% glycerol, 1xTBE and HPLC-water. The separation of the allelic variants was achieved by run conditions at 12 kV for 36 min and by a run temperature at 24°C. The retention times of the allelic variants were identified relative to the ROX size standard. GeneMapper software (version 4.05 Applied Biosystems) was used to process the SSCP data. Peak pattern results were reproducible as they were run 3 × with size standards.

### Microsatellite typing and heterozygosity

Heterozygosity was assessed using six microsatellite markers: Pdo3, Pdo5, Pdo6, Pdo8, Mcyu4 and Ase18 ([55] and references therein). Single microsatellite marker amplifications were run in a T1 thermocycler (Biometra) in a final volume of 12.5 μL including 50 to 100 ng of genomic DNA, 5 pmol of the forward and the reverse primer, 1U DNA polymerase (FirePol), 3 mM MgCl2, 100 μM dNTPs, and 1× PCR Buffer. After an initial denaturation at 95°C for 5 min, 35 amplification cycles were performed with denaturation at 94°C for 30 s, annealing at 58°C for 90 s, and extension at 72°C for 90 s. A final elongation step was conducted at 72°C for 10 min. The fluorescent-label single microsatellite markers were pooled and fragment analysis was performed (Beckman Coulter CEQ8000 automated sequencer). Number of alleles and mean observed heterozygosity for each locus were, respectively, Pdo3: 20 alleles, 0.87; Pdo5: 25 alleles, 0.74; Pdo6: 82 alleles, 0.91; Pdo8: 18 alleles, 0.34; Mcyu4: 30 alleles, 0.80; and Ase18: 22 alleles, 0.91 (consistent with [56]).

### Mate preference experiment

**Figure 3:**
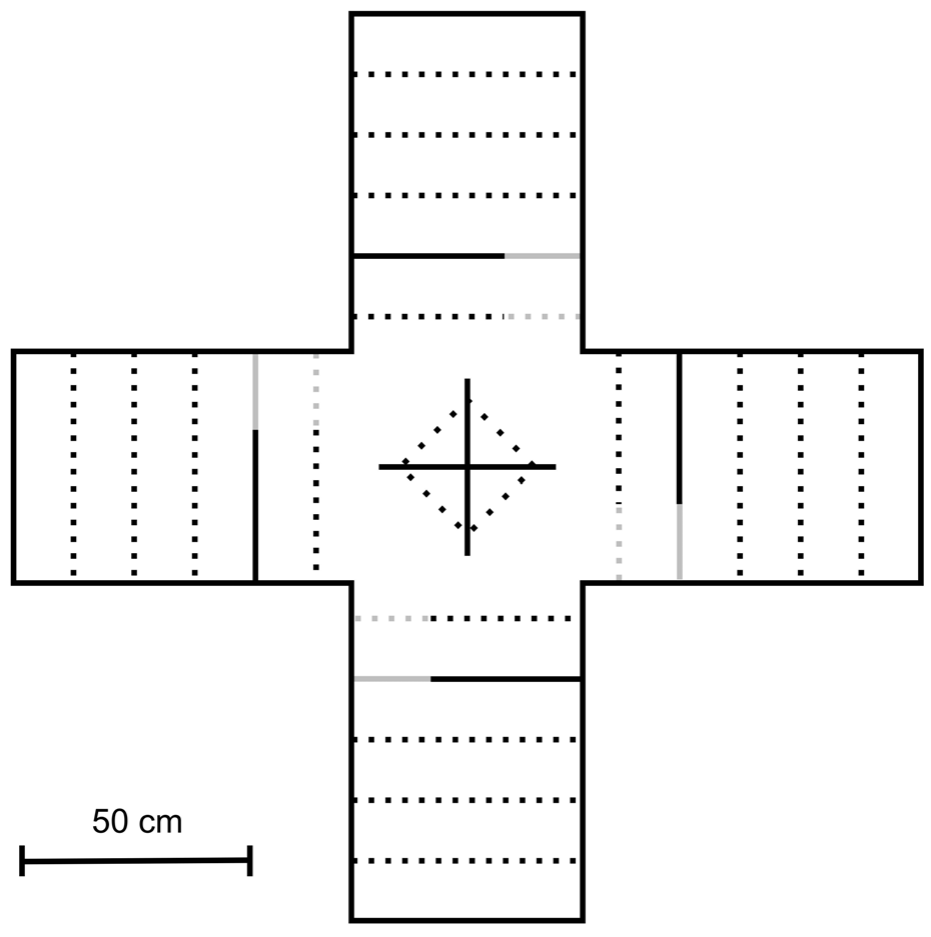
**Schematic overview of the experimental apparatus**. Solid black lines: opaque divisors. Dashed lines: perches. Solid grey line: metal web. Dashed grey lines: part of the perches considered as choice location (choice area).

In April and May we conducted a female mate preference test using an indoor four-choice apparatus (2 m × 2 m × 0.5 m, Figure 3). The apparatus consisted of four choice chambers, separated by opaque dividers, at the four sides of the central choice chamber. An opaque divider was also set up in the middle of the central chamber to avoid visual interaction between the four stimulus individuals (see Figure 3). The central divider also prevented the females from simultaneously observing two or more stimulus. In one corner of the four dividers, an opening (14 × 14 cm) covered by a metal web allowed the female to observe the stimulus in the side chamber. During the experiment the females could see the stimulus through these holes but they could not physically interact. A perch was positioned in front of each of the four chambers. Perches had a line traced, which corresponded to the limit from which a female could observe the stimulus in the nearby compartment (choice area; Figure 3). When the focal female was not present in one of the four perches in front of the opening (choice time), that time was considered "no-choice time". In accordance with the objectives of the study, females had a choice between three males either with low (1-2 alleles), medium (3 alleles) or high (4-6 alleles) number of MHC-I alleles (HM, MM and HM groups respectively). To control for potential position effects, chambers were randomly assigned to the stimulus individuals. As a control, the fourth chamber contained a female (control group, CF group, *n *= 39) to test whether focal females were sexually motivated and did not show a bias among the compartments [57]. Stimulus individuals were tested to three experimental groups of focal females: females with low (LF), medium (MF) and high (HF) diversity (number) of MHC class I alleles (18 different females per group, with groups defined as above for males). We ensured that the stimulus males did not differ in body size (wing and tarsus length and body mass) or black breast patch (badge of status; for more details see [44]) (ANOVA test: all F _2,155 _< 2.23 *P *> 0.12). The experiment consisted of 54 mate-preference trials, each with a different focal female as the subject. All birds were unfamiliar with each other because they came from different visually separated aviaries. At the beginning of a trial, test female and stimulus individuals were placed in their experimental chambers and allowed at least 30 min to acclimate before the trial began. After that period, the opaque separators, that covered the mesh windows, were removed and the position of the female was recorded every 1 s for 1 h (all trials were video recorded and then analyzed by students, blind with respect to the MHC genotype of the individuals). We measured the time spent by a female on the part of the perch in front of a male's compartment, and preference was expressed as the proportion of time in front of each male over the total time in the choice area (e.g. [58-61]). Outcomes from all female preference experiments were analysed with a generalized linear model (GLM) in which female preference was the dependent variable (see also [61]). To test the effect of the interaction between female group and stimulus group on female mate preference we used a GLM post hoc test (Tukey honestly significant difference test; see also [57]). Statistical analyses were performed with SPSS 17.0. All the results are presented as mean ± SE. All tests are two-tailed. Analyses were checked to ensure that they met the assumptions of parametric statistics.

## Authors' contributions

MG, CB and HH conceived the project. MG performed the experiment, analysed the data and fine-tuned the manuscript. DP gave suggestions for genetics. CB performed MHC characterization. All authors contributed to the manuscript, read and approved the final manuscript.
